# Agricultural Extension Messages Using Video on Portable Devices Increased Knowledge about Seed Selection, Storage and Handling among Smallholder Potato Farmers in Southwestern Uganda

**DOI:** 10.1371/journal.pone.0169557

**Published:** 2017-01-25

**Authors:** Bjorn Van Campenhout, Senne Vandevelde, Wilberforce Walukano, Piet Van Asten

**Affiliations:** 1 Development Strategy and Governance Division, International Food Policy Research Institute (IFPRI), Kampala, Uganda; 2 LICOS Centre for Institutions and Economic Performance, KU Leuven, Leuven, Belgium; 3 International Institute of Tropical Agriculture (IITA), Kampala, Uganda; University of Idaho, UNITED STATES

## Abstract

To feed a growing population, agricultural productivity needs to increase dramatically. Agricultural extension information, with its public, non-rival nature, is generally undersupplied, and public provision remains challenging. In this study, simple agricultural extension video messages, delivered through Android tablets, were tested in the field to determine if they increased farmers’ knowledge of recommended practices on (i) potato seed selection and (ii) seed storage and handling among a sample of potato farmers in southwestern Uganda. Using a field experiment with ex ante matching in a factorial design, it was established that showing agricultural extension videos significantly increased farmers’ knowledge. However, results suggested impact pathways that went beyond simply replicating what was shown in the video. Video messages may have triggered a process of abstraction, whereby farmers applied insights gained in one context to a different context.

## Introduction

To feed a growing population, agricultural production needs to increase dramatically. The global population is projected to increase to more than 9 billion by 2050. In order to meet the demand for increasingly calorie-intense and complex diets, overall food production would need to increase by some 70 percent between 2005 and 2050 [1]. However, this increase needs to be accomplished against a background of greater competition for land, water, and energy, and in the context of a changing climate. Therefore, sustainable intensification, whereby modern inputs and improved technologies and practices allow farmers to grow more food from the same area of land while reducing environmental impacts, is imperative [2, 3]. Indeed, in Asia, the use of modern inputs and techniques on a large scale (e.g, inorganic fertilizer and hybrid seeds) and improved technologies (e.g., irrigation and row planting) led to a substantial increase in yields in a relatively short period of time [4]. Conversely, yield gaps, expressed as the difference between actual yields and attainable yields when modern inputs and proper practices are used, remain large in many parts of the world, and especially in Africa south of the Sahara. Reducing these gaps in underyielding locations through the use of modern inputs and improved technologies is expected to boost production, increase food security, and reduce poverty [5].

Use of modern inputs and adoption of recommended farming practices is generally low in developing countries. There are different reasons why smallholder farmers shun agricultural intensification investments. It may be that the agricultural technology is simply not profitable, so nonadoption is a rational response on the part of the farmer. However, more careful investigation suggests substantial heterogeneity, with some farmers adopting certain practices while others do not. Such selective adoption is indicative of various market imperfections [6]. For example, new technologies may affect the type and amount of labor needed, and poorly functioning labor markets may interfere with adoption decisions [7]. Land market imperfections, characterized by insecure property rights, may also prevent some farmers from investing in, for instance, soil conservation measures [8]. New, unfamiliar technologies may carry small downside risk, potentially affecting adoption when insurance markets are incomplete [9]. Other market inefficiencies that have been found to affect technology adoption include input and output market inefficiencies [10, 11], missing credit markets [12], and information market inefficiencies [13].

In poor, remote communities, information market inefficiencies in particular are thought to be important in explaining underadoption: if an individual does not know that a technology exists, does not know about its benefits, or does not know how to use it effectively, then he or she will not adopt the technology. Since information is a public, nonrival good, governments across the developing world have started providing extension services on a large scale to address these information gaps. However, they have done so with mixed success. While most studies have reported positive impacts of extension services, these effects are far from general [14], with cost-effectiveness, limited scaleability and accountability frequently cited as issues [15]. Information and Communication Technologies (ICTs) have been advanced as a promising way to improve agricultural extension services [16]. However, to date, most use of ICTs in development has been in the provision of financial services through mobile phones [17], while the potential for agricultural extension has been studied less.

This research contributes to the literature on innovations in agricultural extension services by testing whether showing simple and short videos is a viable alternative to more elaborate, and hence more costly, extension practices. More specifically, this paper investigates whether simple informational interventions would succeed at increasing knowledge among smallholder farmers. To do so, an experiment was set up among potato (*solanum tuberosum*) farmers in the Kigezi subregion in southwestern Uganda, where poor seed quality is an important reason for low yields. For instance, by using quality seed from a certified source instead of reusing seed from the previous harvest, agronomic data suggested tuber yields could be doubled. While providing access to clean planting material derived from basic foundation seed should remain a key policy priority, current seed systems are too weak to have a significant impact in the short to medium term. Therefore, in a context where farmers rely heavily on saved seed as planting material for the next season, the selection, storage and handling of seeds are important pathways to improve quality. The interventions therefore focused on Positive Seed Selection (PSS), the practice of keeping the best potatoes as seed material [18], and on Proper Seed Storage and Handling (PSSH), involving best practices to preserve seed potatoes between harvesting and planting them in the next season.

The interventions relied on two basic information treatments. Simply providing information has been found to be very effective in changing behavior in a range of applications. For example, Jensen [19] observed that returns on education were perceived much lower than they actually were, and found that students who were told about the higher measured returns complete on average 0.20–0.35 more years of schooling over the next four years than those who were not given this information. Dupas [20] found that providing teenagers with information about the risk of HIV infection relative to one’s partner’s age significantly impacted sexual behavior. In the context of agricultural extension, Cole and Fernando [13] demonstrated that delivering timely, relevant, and actionable information and advice to farmers reduced knowledge gaps and increases productivity.

The information treatments took the form of video messages shown to individual farmers on Android tablets. Video, combining both visual and verbal communication methods, has been found promising in providing low-literacy populations with skills, information, and knowledge on complex technical topics [21–23]. Steady progress in mobile video display equipment and increasing mobile phone penetration and internet connectivity in rural areas have made it easier and cheaper to distribute video content. In addition to increasing knowledge directly, video has also been found to induce behavioral changes in poor countries. While some of this behavioral change may be a consequence of the newly acquired knowledge, research by Bernard et al. [24] suggested that videos featuring role models can induce behavioral change by affecting the motivation and aspirations of farmers.

The objective of this study was to investigate if short agricultural extension information messages, delivered to individual farmers through Android tablet computers, increased farmers’ knowledge. The method used was a field experiment where farmers were randomly allocated to different treatment groups. One treatment consisted of farmers being shown a video on how to select the best potatoes as seed materials to be used for the next planting season. Another treatment consisted of farmers being shown a video on how to store potatoes to be used as seed materials for the next season. We also included a control group in our experiment. After the treatments were administered, knowledge about seed selection and seed storage, obtained through a short quiz, was compared between treatment and control groups to find out if the videos increased farmers’ knowledge.

## Materials and Methods

### Experimental Units

Baseline data was collected from potato farmers in three districts (Kisoro, Kanungu, and Kabale) in southwestern Uganda for the 2013/2014 agricultural season. The three districts together accounted for about 47 percent of total potato production in Uganda [25]. With the assistance of the Uganda Bureau of Statistics, 35 enumeration areas were randomly selected. Within each enumeration area, all households were listed and it was determined whether they were growing potatoes. From these lists, potato farmers were randomly selected to be interviewed on a range of socio-economic variables. These variables included household composition, land holdings and use, assets, crop production, and consumption expenditure. In addition, detailed baseline information was collected related to potato farming, including experience, extension received, knowledge of recommended practices, and inputs and methods used. For the actual experiment, 248 farmers were selected from this baseline (see next section).

Ethics approval for this research was obtained from the International Food Policy Research Institute’s Institutional Review Board (IRB #00007490 FWA #00005121). Consent of each selected farmer was obtained orally. After the enumerator introduced him or herself, the aim of the study and procedures were explained and it was made explicit participation was voluntary. The farmer was then asked to agree to participate and this was recorded on the questionnaire form by the enumerator. All this was done in the local language of the farmer by a trained enumerator. The decision to obtain oral instead of written consent was motivated by the fact that many of the farmers in the study were illiterate. It was felt that insisting on written consent might lead to a feeling of shame or frustration on the part of the illiterate farmer. This procedure was approved by the IRB on February 2nd, 2016 (IRB Approval Number 2016-12-DSGD-M).

### Experimental Design

The 248 selected farmers were randomly allocated to different treatment conditions within a 2 x 2 full factorial design. In particular, 124 farmers received the first treatment (Positive Seed Selection, or PSS see “Treatments” subsection below) and 124 farmers received the second treatment (Positive Seed Storage and Handling or PSSH). However, half of this last subset overlapped with half of the farmers that were treated with PSS. As such, 62 farmers received both PSS and PSSH treatments, and 62 farmers received no treatment at all. The full factorial experiment was designed to test two main effects (PSS and PSSH) and consisted of four different treatment conditions (Control, PSS, PSSH, PSS + PSSH). Such designs are very efficient, as it allows one to use the entire sample to test main effects. That is, to test if the PSS treatment worked, 124 farmers that received the PSS treatment can be compared to 124 farmers that did not receive the PSS treatment. Similarly, to test if the PSSH treatment worked, 124 farmers that received the PSSH treatment can be compared to 124 farmers that did not receive the PSSH treatment. Apart from testing main effects, factorial designs also allow estimation of interaction effects, comparing for instance the subgroup of farmers that received only PSS to the subgroup of farmers that received both PSS and PSSH. However, when testing interactions, sample size reduces as there are now only 62 farmers in each experimental condition. As the sample size of this study was determined on the basis of testing main effects only, testing interactions is likely to suffer from a lack of statistical power and so interactions were not considered in this study.

Instead of simple randomization, an ex ante matching procedure was used, where farmers that were similar along a range of characteristics were matched into blocks prior to randomization. In particular, a farmer was selected randomly and matched to another farmer that was most similar to this first farmer on the basis of a set of characteristics (see below for the list of characteristics). To find the most similar farmer, the square root of the sum of squared standardized differences of the measures for these characteristics was minimized. This was repeated three times and the resulting group of four farmers was given a unique block number and removed from the sample. This procedure was then repeated until all farmers were matched in groups of four farmers. Finally, the four different treatment conditions from the factorial design (Control, PSS, PSSH, PSS + PSSH) were randomly allocated to the four farmers within each block. As the sample size was 248 observations, this resulted in 62 blocks of four farmers randomly distributed over the four treatment conditions.

This matched procedure guaranteed that farmers that are similar in some characteristics, for instance income, were allocated to different treatment groups. For instance, it reduced the chance that a disproportionate number of high income farmers were allocated to a particular treatment, which would make it difficult to differentiate between the treatment effect and a potential income effect. It is argued that, especially in small samples, such randomization procedures based on matching and stratification can significantly improve on statistical power [26]. The procedure was also described in detail in the pre-analysis plan, which is available at the American Economic Association’s registry for randomized controlled trials.

Matching was performed on ten characteristics. All characteristics received an equal weight in the objective function. Three variables related to household demographics that are standard in empirical specifications of agricultural household models were included [27]. Household size is an important measure of human capital, particularly in the smallholder agricultural settings with imperfect labor markets [28]. The age of the household head was included to capture experience and life-cycle effects. Observations were also matched on gender of the household head, as previous knowledge about modern agricultural techniques and inputs may differ by sex [29]. The area of potatoes grown, as well as a variable that indicated whether the household received extension on potatoes in the past, the logarithm of potato yields, and the logarithm of welfare per capita were also all included in the matching procedure. Other variables included travel distance to the closest farm input dealer or farm supply store and access to credit.

This research adhered to the highest standards in terms of transparency. For instance, a pre-analysis plan, developed before the interventions, detailed what sampling methods would be used, what specifications would be run, and what outcome variables would be analyzed. The pre-analysis plan also included orthogonality tests for a range of relevant baseline characteristics and demonstrated there are no ex ante systematic differences between treatment and control groups. The experiment was also registered at the American Economic Association’s registry for randomized controlled trials (https://www.socialscienceregistry.org/trials/1014). Finally, the entire project, including all data which is also accessible as S1 Data S1 Data, computer code, and documents, was under revision control using Git (https://git-scm.com/), a free and open source distributed version control system, and mirrored on an online repository (https://bitbucket.org/bjvca/potseedrct). This means all material can be downloaded to rerun the analysis and track changes to the project over time, resulting in a level of transparency that is exceptional in the social sciences.

### Inference

The matched randomization procedure was likely to introduce dependence among outcome variables within blocks, so it was necessary to account for clustering during inference [26]. The typical approach to deal with this, using cluster-robust standard errors, is known to be biased in small samples. In addition, to facilitate comparison between simple means comparisons and more complicated models that control for additional variables such as previous knowledge, linear probability models were used. Standard inference becomes invalid when Ordinary Least Squares (OLS) is used with binary outcome variables. To address these two issues, randomization inference (RI) was used, as RI remains exact regardless of the regression specification [30]. Instead of relying on a theoretical distribution, RI involves comparing the test statistic with the distribution of the test statistic under each possible allocation of treatments. In this study, only the main effects in the factorial design were tested. In particular, outcomes of 128 farmers that received PSS were compared to outcomes of 128 farmers that had not received PSS. Similarly, outcomes of 128 farmers that received PSSH were compared to farmers that had not received PSSH. RI thus involved computing outcomes within each block of four farmers for all 6 possible permutations of treatment (T) and control (C): {(T,T,C,C),(T,C,T,C),(T,C,C,T),(C,C,T,T),(C,T,T,C),(C,T,C,T)}. For 62 blocks, this led to a total of 6⌃62 permutations, so instead of actually computing all combinations, inference was based on a random subset of 10,000 permutations.

Not accounting for the method of randomization might have resulted in overly conservative standard errors and a significant reduction in power [26]. Therefore, in addition to accounting for clustering through the use of RI, regression models including fixed effects at the block level were also run. Recall that in each block *b* = {*b*_1_, …, *b*_62_}, four experimental conditions (Control, PSS, PSSH, PSS + PSSH) were randomly assigned. As such, to test a main treatment effect in our study, each block always had two control (*C*) and two treated (*T*) observations *t* = {*C*_1_, *C*_2_, *T*_1_, *T*_2_}. For example, to test the main treatment effect of the PSS treatment, within each block, one farmer that received the PSS treatment and one farmer that received both the PSS treatment and the PSSH treatment were compared to a farmer that received only the PSSH treatment and a farmer that received no treatment at all. Similarly, to test the main treatment effect of the PSSH treatment, within each block, one farmer that received the PSSH treatment and one farmer that received both the PSSH treatment and the PSS treatment were compared to a farmer that received only the PSS treatment and a farmer that received no treatment at all. Average treatment effects (*β*) were estimated by regressing the outcome variable (*y*) on the treatment indicator *I* where *I* = 1 *if* *t* = {*T*_1_, *T*_2_} and 0 otherwise, and on fixed effects for the blocks (*δ*_*b*_):

$$y_{t,b}=\alpha +\delta_{b}+\beta I_{t,b}+\varepsilon_{t,b}$$ (1)

where *α* was a constant and *ε*_*t*,*b*_ was an error term. Since an overall constant was included in the model, only 61 fixed effects were included.

### Treatments

The first treatment consisted of a video on Positive Seed Selection (PSS), see S1 Video in S1 Video. In this video, a potato farmer from the area introduces himself and explains that his experiments over the years have taught him that good-quality planting material is key to becoming a successful farmer (0:00–1:25 minutes into the video). He illustrates the benefits of good quality seed by contrasting healthy fields, plants, and tubers to diseased ones (1:25–2:35 minutes). The farmer also explains how he used to do it wrong, pulling out the strongest plants first to eat or sell, and hence was left with small and malformed tubers for planting. He explains how this quickly leads to seed degeneration (2:35–3:19 minutes). Next, the concept of Positive Seed Selection is introduced (3:19–4:26 minutes). In particular, the farmer explains that, at time of flowering, the tallest plants with at least four stems should be marked and pegged for follow-up. Pegs (in the form of wooden sticks in the video) should be removed when plants get diseased or when they grow slowly (4:26–5:05 minutes). At the time of harvest, pegged plants should be harvested first (5:05–5:16 minutes). Only egg sized tubers should be retained for planting material (5:16–5:27 minutes). Tubers should look healthy, without cuts or bruises, and it is advised to only keep tubers with at least four eyes (5:27–5:43 minutes). The video ends by recapitulating the most important components of Positive Seed Selection. (5:43–7:02 minutes). This video was produced in both Rufumbira and Rukiga, the two languages spoken in the study area.

The second treatment consisted of a video on Proper Seed Storage and Handling (PSSH), see S2 Video in S2 Video. The first part, in which a farmer introduces himself as a successful potato grower from the region and illustrates the benefits of good-quality seeding material by contrasting healthy fields, plants and tubers to diseased ones, is similar to the first part in the video used in the PSS treatment (0:00–2:08 minutes into the video). The farmer also explains that he used to store and handle seeds incorrectly, storing potatoes in sacks or together with other crops in places that were too dark and inadequately ventilated (2:08–3:24 minutes). The farmer then introduces PSSH. He first underscores potatoes should be spread out on wooden racks, or on dried grass on the floor (3:24–4:03 minutes). Second, seed potatoes should be stored in a separate room, away from animals and humans (4:03–4:11 minutes). Third, seed potatoes should be stored in a well ventilated place in diffuse lighting conditions and checked regularly for rotten tubers (4:11–5:07 minutes). Finally, the farmer advises the use of a cheap organophosphate insecticide for seed preservation and underscores the importance of cleaning all tools used during seed production to avoid contamination (5:07–5:45 minutes). Like the PSS video, this one ends by summarizing the most important aspects of PSSH (5:45–7:05 minutes). This video was also produced in both Rufumbira and Rukiga languages. The choice of which techniques and information to highlight in both treatments was based on extensive interviews with potato-growing experts (seed producers, extension officials, agronomists) in the area and on analysis of data previously collected among potato farmers.

The treatments were administered at the individual level. Enumerators were trained to create a discreet environment. They were instructed to find a quiet place where they were unlikely to be disturbed. Spouses and other family members (apart from children) were politely asked to leave for the duration of the interview and the screening of the videos to maintain optimal concentration. Most of the treatments were administered in the home of the farmer or in the field.

Individual treatments are uncommon in agricultural extension. This is because it is often too costly to send agricultural extension workers to each individual farmer. Working with groups may also be more effective, as farmers may engage in discussion with each other and ask clarification. However, it may also result in group dynamics that are difficult to control by the experimenter. At a more practical level, it is very difficult to exclude people from group screenings at community centers, greatly complicating the randomization. Showing videos on Android tablets has a much lower marginal cost, making it a potentially important technology to reach remote farmers.

### Outcomes

To test whether the videos reduced farmers’ knowledge gaps, farmers were asked to take a short quiz. In particular, farmers were subjected to six multiple choice questions. All participants, including control farmers, were presented with all six questions. The questions were asked after the video was shown, unless for control subjects, who were given the six questions without any additional information. Each question had three possible answers, which were read out to the farmer, and only one answer was correct. The farmer was then instructed to indicate what he or she thought was the correct answer. The answer of the farmer was then indicated by the enumerator on the questionnaire. Two of the six questions were related to topics that were discussed in the PSS treatment. As such, it was expected that farmers who received the PSS treatment would do better in answering these questions correctly. Another two questions were related to topics covered in the PSSH treatment, and it was expected that farmers who were provided information in the PSSH treatment to answer these questions correctly. A final set of two questions, one on a topic related to PSS and one on a topic related to PSSH but not explicitly covered in the treatment videos were also included. It was expected that the incidence of correct answers to these two “control” questions would not differ between the treatment and control farmers.

All multiple choice questions and the correct answers are summarized in Table 1. The two questions that test knowledge provided by the PSS treatment related to which plants to peg and the size of the tuber to select as planting material. The answers will be referred to in the analysis below by variable names *sel1* and *sel2* respectively. To test the effectiveness of the PSSH treatment, the first question related to which lighting conditions seed potatoes should be stored in. The second question asked how seed potatoes should be stored. The answers to these questions were recorded in variables called *store1* and *store2* respectively.

**Table 1:** Multiple choice questions used test effect of treatments on knowledge.

code	question	answer (correct one underlined)		
sel1	Which plants should you peg for seed selection?	The largest plants in the field that look healthy	The smallest plant in the field that look healthy	Average sized plants that look healthy
sel2	What size should a potato seed tuber be?	The larger the better	Size of an egg	The smallest ones you find
store1	Where do you store your seed potatoes?	In direct sunlight	In a dark place	In indirect light (diffuse light)
store2	How should you store your seed potatoes?	In bags that have been thoroughly cleaned with JIK	Spread out on racks or on dried grass on the floor	In airtight containers or buckets with a closing lid
gen1	Which of the following statements is correct?	Immediately after harvest, you should thoroughly wash potato seeds before putting them in storage using JIK	Immediately after harvest, you should thoroughly wash potato seeds before putting in storage using clean water	You should never wash potato seeds before putting them in storage
gen2	When picking a field for positive seed selection…	Pick a garden that is in highlands and in isolated areas	Pick lowlands with plenty of water	Pick a garden close to your house or in densely populated area

Of the questions on topics not explicitly covered in either the PSS or the PSSH videos, the first asked respondents to indicate which of three statements was correct. All three alternatives related to how potato seed should be treated immediately after harvest and before putting them in a store. While this knowledge was not explicitly covered in the PSSH video, it was related to Proper Seed Storage and Handling. The answers to this question were recorded as variable *gen1*. The second question asked if farmers knew where fields for seed potato production should ideally be located. While this knowledge was not explicitly covered in the PSS video, it could be categorized under Positive Seed Selection knowledge. The answers to this question were recorded as variable *gen2*.

## Results

**Fig 1:**
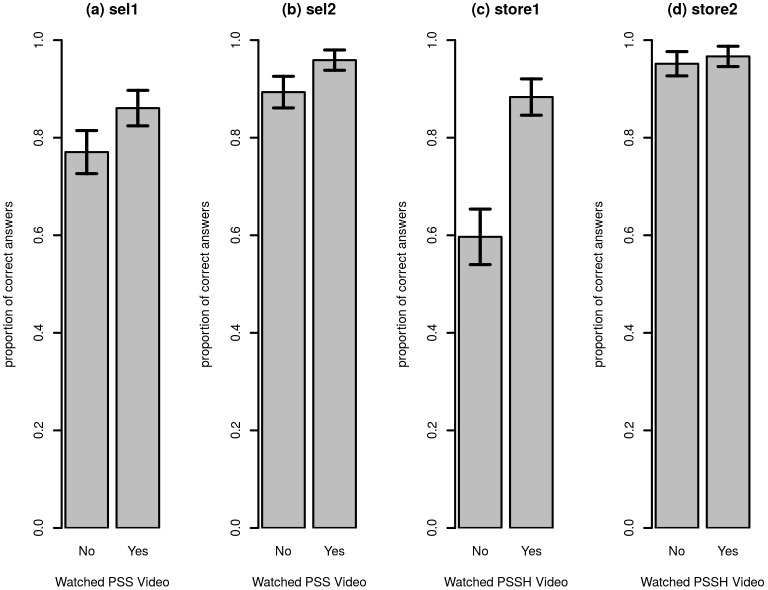
Effect of PSS and PSSH treatment on knowledge covered in videos. Figure notes: 10 percent error bars are included.

Showing extension videos on Positive Seed Selection (PSS) to farmers increased their knowledge related to seed selection practices covered in the video. The first bar chart in Fig 1-a, shows the proportion of farmers that knew the largest healthy-looking plants need to be pegged for follow-up for seed selection (*sel1*). The figure revealed that about 77 percent of farmers who were not shown the PSS video were able to indicate the correct option in the multiple-choice question. Among farmers who were shown the PSS video, this proportion increased to 86 percent. The increase was statistically significant (one-sided RI, p = .041). Similarly, the second bar chart in Fig 1-b, shows that the proportion of farmers who knew that egg-sized tubers are the best seeding material was 89 percent among those who had not received the PSS treatment and 96 percent among those who were shown the video. Again, the increase in knowledge was statistically significant (one-sided RI, p = .042).

Likewise, showing videos on Proper Seed Storage and Handling (PSSH) increased knowledge about recommended storage and handling practices covered in the video. As indicated by the third bar chart in Fig 1-c, only about 60 percent of participants who were not shown the PSSH video knew that seed potatoes needed to be stored in diffuse light (*store1*). This proportion increased to 88 percent among farmers who had seen the video on PSSH. The increase in the proportion was statistically significant (one-sided RI, p<.001). Finally, as shown in Fig 1-d, 95 percent of farmers who were not shown the PSSH video knew that seed potatoes should be spread out on racks (*store2*). This percentage increased to 97 percent among farmers who had seen the PSSH video, but the increase was not significant (one-sided RI, p = .372). However, to reduce the influence of outcomes with limited variation, it was specified in the pre-analysis plan that variables for which 95 percent of observations were the same value would be discarded.

**Fig 2:**
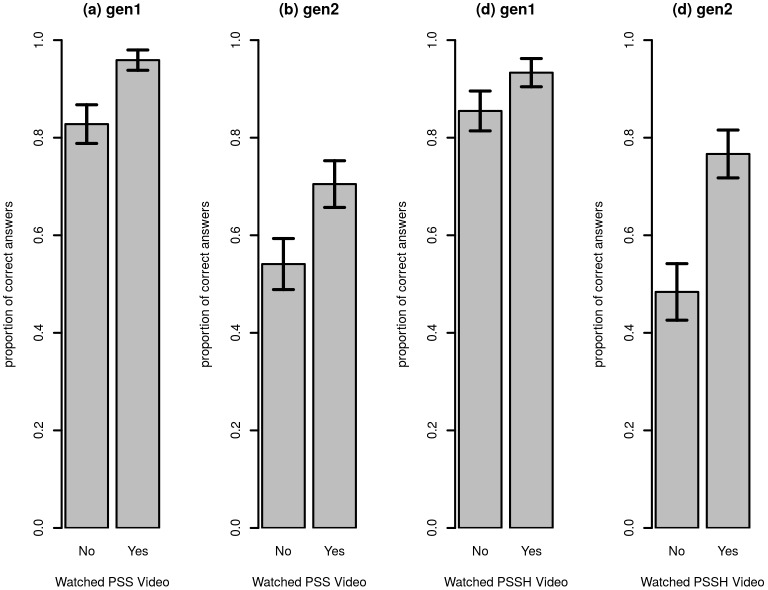
Effect of PSS and PSSH treatment on knowledge not explicitly covered in videos. Figure notes: 10 percent error bars are included.

Being shown any video had the potential to increase knowledge beyond what was explicitly covered in the video. For example, the first bar chart in Fig 2-a shows that farmers who received the PSS treatment were also significantly more likely to know that seed potatoes should not be washed before being stored (one-sided RI, p<.001). The second bar chart, Fig 2-b, shows that the same treatment also significantly increased the likelihood of a farmer knowing that fields for planting materials should ideally be located in highlands, away from human settlements (one-sided RI, p = .006). The third bar chart, Fig 2-c, shows that farmers exposed to the PSSH treatment were also more likely to know that seed potatoes should not be washed before being stored (one-sided RI, p = .032), and the fourth, Fig 2-d, shows they also knew fields for planting materials should ideally be located in highlands (one-sided RI, p<.001). This effect was also present when outcomes on knowledge related to one treatment were compared between groups based on the other treatment: farmers who received the PSS treatment score significantly better on *store1* and *store2* than those who had not, and farmers who received the PSSH treatment score significantly better on *sel1* and *sel2*.

To account for the clustering due to the block matching procedure, regressions using a within-block specification were also run (see Eq 1). The estimates of the average treatment effects can be found in Table 2 and were consistent with the findings in Figs 1 and 2. For instance, the PSS treatment induced an increase of 9 percentage points in the proportion of farmers who answered correctly on the first seed selection knowledge question. This was exactly what was found in the simple means comparisons above, but the standard error was slightly higher, leading to a p-value of.05. The effect of PSS on the second question related to seed selection was also estimated at 7 percentage points, and this time the inclusion of block fixed effects reduced the standard error. Also consistent with the means tests, the PSSH treatment had a large and significant effect on knowledge related to storage as measured by the first question, while the effect on the second question was not significant (probably due to limited variation in the outcome variable).

**Table 2:** Effect of PSS and PSSH treatments on knowledge.

	sel1	sel2	store1	store2	gen1	gen2
PSS	0.09 [0.050]	0.07 [0.012]	0.28 [0.000]	0.02 [0.165]	0.13 [0.000]	0.17 [0.002]
PSSH	0.07 [0.091]	0.08 [0.003]	0.29 [0.000]	0.02 [0.163]	0.08 [0.010]	0.28 [0.000]

Table notes: one-sided p-values are in square brackets and were based on randomization inference, with a random sample of 10,000 permutations used. All regressions included 61 block fixed effects and a constant. PSS = positive seed selection; PSSH = proper seed storage and handling.

Again consistent with Figs 1 and 2, it was found that showing a video not only increased knowledge related to what was featured in the video, but there seemed to be a more general knowledge effect. For example, as a result of being shown either of the videos, an increase was registered in the proportion of farmers who answer correctly on the questions that were not explicitly covered in the video (*gen1* and *gen2*). Also, farmers who were shown the PSS video scored higher on *store1*, a question about PSSH practices. Similarly, the likelihood of a farmer correctly answering *sel1* and *sel2*, questions on PSS, increased by 7 and 8 percentage points, respectively, as a consequence of having been shown the PSSH video.

The results in Table 2 may mask heterogeneity in the average treatment effect. In particular, it may be that farmers with little prior knowledge about PSS, PSSH, or both were more likely to benefit from the information that was contained in the videos. To test this hypothesis, information collected during the baseline was used. In particular, information on farmers’ reported awareness of the importance of using clean and disease-free planting materials was added in a regression to control for previous knowledge related to PSS. In addition, information on farmers’ awareness that seeds should be stored on dried grass in the shade was used to control for previous knowledge related to PSSH. This was done in the regression framework of Eq (1) by interacting both of these variables with the treatment indicators. Results are summarized in Table 3. Because the study was not designed to identify heterogeneous treatment effects, and thus sample size is likely to be too small, attention is confined to the variables that display the most variation (*sel1*, *store1* and *gen2*).

**Table 3:** Effect of PSS and PSSH treatments on knowledge, controlling for previous knowledge.

	sel1	store1	gen2	gen2	sel1	store1
PSS	0.31 [0.017]		0.13 [0.203]			0.36 [0.011]
PSSH		0.36 [0.011]		0.32 [0.022]	0.24 [0.055]	
knowPSS	0.09 [0.217]	-0.04 [0.778]	0.05 [0.120]	-0.10 [0.773]	0.08 [0.288]	0.06 [0.249]
knowPSSH	0.12 [0.055]	0.00 [0.138]	-0.23 [0.743]	-0.08 [0.141]	0.09 [0.104]	-0.07 [0.376]
knowPSS*PSS	-0.12 [0.832]		-0.14 [0.831]			-0.07 [0.689]
knowPSSH*PSS	-0.17 [0.862]		0.17 [0.176]			-0.05 [0.622]
knowPSS*PSSH		0.05 [0.358]		0.05 [0.377]	-0.11 [0.808]	
knowPSSH*PSSH		-0.15 [0.821]		-0.08 [0.671]	-0.13 [0.800]	

Table notes: one-sided p-values are in square brackets and were based on randomization inference, with a random sample of 10,000 permutations used. All regressions included 61 block fixed effects and a constant. PSS = positive seed selection; PSSH = proper seed storage and handling.

The first column in Table 3 shows that controlling for prior knowledge substantially increased the treatment effect for the PSS treatment on *sel1*. Among farmers who were not previously aware of the importance of using clean planting materials and who did not know that seed potatoes need to be stored on dried grass in the shade, the coefficient estimate was higher than for those who did have prior knowledge as reported in Table 2 (0.31 compared to 0.09). The second column shows a similar response of storage-related knowledge to the PSSH video. Here, the treatment effect increased from 0.29 (see effect of PSSH on *store1* in Table 2) to 0.36 among farmers who reported having no prior knowledge pertaining to potato seed quality. Contrary to what was found in Table 2, showing the PSS video did not seem to affect knowledge on seed selection not explicitly covered in the video among farmers who had no prior knowledge on the importance of seed quality. However, columns 4 to 6 suggest that even after controlling for previous knowledge, a particular treatment may still increase knowledge about a subject not explicitly covered in the video.

## Discussion

In this study, it was found that showing simple agricultural extension videos to individual potato farmers on portable devices significantly increased knowledge related to seed selection and seed storage and handling among potato farmers. In particular, showing a video that explained Positive Seed Selection (PSS) increased the likelihood that farmers knew about the methods explained in the video. Similarly, showing a video that demonstrated Proper Seed Storage and Handling (PSSH) increased the likelihood that farmers knew about the information explained in the video. This finding suggests that agricultural extension videos are an effective tool for accurate transmission of homogeneous information from a technical source to a low-literacy population, for instance when a technical expert or high-quality trainer is not available or too expensive.

In addition to a direct effect, it was also found that showing a video displaying methods related to a particular aspect of potato growing increased knowledge in general. For instance, showing a video about PSS methods increased knowledge related to seed selection that was not explicitly shown in the video, such as the ideal location for the seed production field. Similarly, showing a video about PSSH increased knowledge related to storage and handling not covered in the video, such as the importance of keeping potatoes dry. Even more, showing a video on one topic, for instance on Seed Selection, increased knowledge in another topic, such as Seed Storage and Handling.

There may be different reasons for such indirect effects. First, they might have been be due to poor design of the treatments. One treatment might have inadvertently contained information that gave the farmer clues about knowledge explicitly shown in the other treatment (and thus associated with the other treatment). For example, when the PSS video explained that tubers should look healthy and one should keep only tubers with at least four eyes (5:27–5:43 minutes into the PSS video), the farmer in the video was shown to be selecting from potatoes that were stored on racks in diffuse light. This might have given viewers clues about proper storage and handling, increasing their likelihood of picking the correct option for the question that was intended to measure the information given on PSSH (*store2*).

Second, the above may suggest that farmers went beyond simply repeating what was shown in videos, and engaged, to some degree, in a process of abstraction (learning concepts from examples) whereby they applied insights gained in one context in a different context. For example, recommending that potato seeds be stored away from other crops, animals, and humans, as was done in the PSSH treatment (4:03–4:11 minutes into the PSSH video), may have raised farmers’ awareness about abstract concepts of hygiene and separation in the context of seed potatoes. This in turn might have prompted farmers to pick the answer from *gen2* most in line with those concepts, namely that the potato seed field should ideally be in a remote place high in the mountains. In cognitive psychology, this type of learning is known as schema abstraction, which posits that knowledge is an abstraction of different memory traces, each representing a specific experience in our lives [31]. In this sense, the videos can be interpreted as experiences that teach farmers something about relevant concepts in their profession.

Third, and perhaps most interestingly, it may be possible that farmers already possessed some of the information needed to identify the correct alternative in the multiple-choice questions, but that a video was needed to trigger the farmer to actually use this information when confronted with the multiple choice questions. For instance, it might have been that a farmer was aware of the recommended practice (through having received extension services in the past for instance), but based his or her actual response on what the customary practice was. Being shown a video in which a fellow farmer talked about the virtues of a range of modern techniques may have served as a visual and auditory cue for the information the farmer already possessed. This finding is consistent with knowledge about the cognitive capacities of human beings [32]. In particular, it suggests the usefulness of video messages to trigger associative recognition and cued recall, which involves retrieval of memory or recognition of previously encountered events, objects, or people with the help of cues and associations [33, 34]. Alternatively, being shown a video might have confirmed the knowledge the farmer had, making him or her more confident to use it. In this way, a simple reminder might both validate information a farmer had but had not applied, or might have served to make it more salient. This is consistent with the finding that receiving information from a trusted source positively affected take-up of rainfall insurance among smallholders in India [35].

This last explanation, the apparent activation of latent agricultural knowledge by showing short videos to farmers, is undoubtedly the most interesting outcome of this study and deserves to be explored more in detail. It seems to suggest that, while videos can be of value in itself (and especially in contexts where it is difficult or prohibitively costly to implement full-scale extension services), they work best when complementing previously acquired extension information. This could potentially have considerable implications for the way in which extension services are organized in the developing world today. In addition, the insights emanating from this paper represent an important addition to the agricultural technology adoption literature.

First, since the results indicated the importance of latent knowledge, it could be argued that videos will be most effective in contexts where farmers already have some notion about the techniques explained. They could have acquired this information through extension services, through interaction with experts or simply through word-of-mouth. This suggests that videos will work best when they deal with traditional crops for which the information base is unavoidably larger, rather than for newly introduced crops or varieties. Since it has been argued that extension efforts should especially be focused on new techniques and crops [36], there has been a tendency to overemphasize those in practice. However, the results in this paper highlight the importance of not neglecting profitable existing, traditional or simple techniques that simply require further reminders and encouragement. In that sense, this is consistent with the findings in [37], who showed that farmers “fail to notice” some important elements in the information they already possess. As has been shown here, videos may be the perfect tool to bring out that knowledge and make farmers more confident in applying it.

Second, the results in this paper demonstrated that videos could be effective in reinforcing previously acquired knowledge or beliefs, but that they might be less suited to deal with existing, but unprofitable or even harmful traditional beliefs and superstitions. Previous studies have shown that providing people with information that contradicts their beliefs might actually do more harm than good. For instance, in the context of health care, [38] described the failed diffusion of boiling drinking water in a village Peru simply because of the negative association between hot water and illness. So, in designing an intervention, one should always be considerate of the type of knowledge already present at the local level. If it is in line with the recommended practices, videos (or other simple methods) might represent a viable alternative to more costly extension services. However, if beliefs have to be changed, more extensive methods are probably required.

Third, social networks matter. This study had the benefit of being able to target farmers directly and individually, thus allowing the pure knowledge effect of the video to be captured. However, when such an intervention would be scaled up, this would not be feasible nor desirable. In most instances, groups of farmers would be shown the video at the same time, unleashing social interactions which might reinforce or reduce the impact of the video. As such, it is crucial to consider the social context in which the intervention will take place. For instance, when a certain community is organized along strict hierarchical lines, it is instrumental to get the leaders on board. If their beliefs or agenda do not coincide with the information provided, videos will not be effective (see also previous paragraph). The importance of social networks in agricultural knowledge adoption is illustrated by the expanding experimental literature on the subject [39–41].

Finally, this study focused on the use of videos to transmit agricultural knowledge, but in principle, there exist many more means to achieve the same objective. One can think of radio messages, text messages [42] or even educational posters. For these methods to work, the message should be clear, concise, easily understood and, perhaps most importantly, applicable to a heterogeneous audience of farmers. When the information or the technique requires a far-reaching degree of customization (as has been found to be the case for many new innovations [43]), standardized methods will not suffice. Again, a consideration of the social context is crucial to decide upon the form of the intervention. There should be sufficient openness towards the presented technique in the community, otherwise, the intervention will fail to achieve its objective.

The findings from this study suggest that videos are likely to become an indispensable part of the agricultural extension tool kit, sometimes replacing, but in most cases complementing traditional extension services. They also suggest that specific videos aimed at transferring narrow technical information are effective. In addition, video messages that show more general information, such as the importance of nutrient management or general hygiene to combat pests and diseases, may even be more important. Such videos may provide visual and auditory stimuli that lead to cognitive processes of schema abstraction and cued recall, which, in contrast with classic studying, has the potential to create new and longer-lasting connections between concepts [44]. In addition, this research suggests that videos should be crop and context specific, featuring model farmers to maximize the potential of videos to leverage knowledge farmers already possess but may not be confident enough to use. As such, every intervention should be preceded by an agronomic analysis as well as a study of the existing hierarchies and social networks in the communities in which the intervention will take place.

## Supporting Information

S1 Data This file provides that data that was used in this study. The first column provides the treatment that the farmer received (Sel+Store, Store, Sel or Ctrl). The next six columns show if the participants to the study answered correct (TRUE) or not (FALSE) on the respective multiple choice question. The next column indicated the block number. The final two questions indicate if subjects had previous knowledge related to seed selection, and storage and handling. The data can be found on https://www.dropbox.com/s/2dtuz8e9fl2m4ba/data_PlosOne_VanCampenhout2016.csv?dl=0.
(CSV)

S1 Video PSS treatment. The video that was used for the first treatment can be found on https://www.dropbox.com/s/7o5zcrlr1hvw3m7/moviePSS.mp4?dl=0. This is the Rufumbira version. A version of the same video was also produced in the Rukiga language. This video can be found on https://www.dropbox.com/s/xt5uahqa02m3vmq/moviePSSRukiga.mp4?dl=0.
(MP4)

S2 Video PSSH treatment. The video that was used for the second treatment in the Rufumbira language can be found on https://www.dropbox.com/s/bg0ks15uomr70m9/moviePSSH.mp4?dl=0. The version in Rukinga is available from https://www.dropbox.com/s/nqnm6s03gyw43iw/moviePSSHRukiga.mp4?dl=0.
(MP4)
